# Extranodal Marginal Zone Lymphoma: Integrating Etiology, Microenvironment, and Genetics Into Clinical Decision‐Making

**DOI:** 10.1111/ejh.70245

**Published:** 2026-06-23

**Authors:** Mamdouh Skafi, Santino Caserta, Enrica Antonia Martino, Ernesto Vigna, Antonella Bruzzese, Nicola Amodio, Marco Fiorillo, Eugenio Lucia, Graziella D'Arrigo, Virginia Olivito, Caterina Labanca, Francesco Mendicino, Maria Eugenia Alvaro, Giovanni Tripepi, Fortunato Morabito, Massimo Gentile

**Affiliations:** ^1^ Emergency and Internal Medicine Department Saint Joseph Hospital East Jerusalem Palestine; ^2^ Hematology Unit, Department of Onco‐Hematology AO of Cosenza Cosenza Italy; ^3^ Department of Experimental and Clinical Medicine University of Catanzaro Catanzaro Italy; ^4^ Department of Pharmacy, Health and Nutritional Science University of Calabria Rende Italy; ^5^ Istituto di Fisiologia Clinica del CNR di Reggio Calabria Consiglio Nazionale Delle Ricerche Reggio Calabria Italy; ^6^ AIL Sezione di Cosenza Cosenza Italy

**Keywords:** antigen‐driven lymphomagenesis, extranodal marginal zone lymphoma, NF‐κB signaling, pathogen‐directed therapy, precision treatment strategies

## Abstract

Extranodal marginal zone lymphoma (EMZL) represents a unique paradigm among indolent B‐cell neoplasms, in which lymphomagenesis is frequently driven by chronic antigenic stimulation within tissue‐specific microenvironments. Persistent infectious or autoimmune triggers promote the development of ectopic lymphoid tissue and sustain B‐cell activation, while recurrent genetic alterations—most prominently those that converge on NF‐κB signaling—enable progressive escape from antigen dependence. This dual biological foundation explains both the indolent clinical course of most cases and the remarkable therapeutic vulnerability of early‐stage, infection‐driven disease. Clinically, EMZL arise across a wide range of anatomical sites, each characterized by distinct etiologic associations and patterns of progression. As a result, management strategies must be tailored to disease site, stage, and biological context, with a consistent preference for the least intensive intervention capable of achieving durable disease control. Pathogen‐directed antibiotic therapy and involved‐site radiotherapy can be curative in localized disease. In contrast, systemic anti‐CD20‐based immunochemotherapy and targeted agents are reserved for disseminated, refractory, or biologically autonomous lymphomas. Recent advances in molecular profiling, functional imaging, and response assessment are refining risk stratification and treatment selection, shifting therapeutic goals toward early depth of response and quality of life rather than maximal cytotoxic intensity. EMZL thus serves as a model for biologically informed, precision‐oriented management of indolent lymphoid malignancies.

## 1 Introduction

Extranodal marginal zone lymphoma (EMZL) is the most common entity of marginal zone lymphoma (MZL), an indolent group of mature B‐cell neoplasms accounting for approximately 5%–15% of non‐Hodgkin lymphomas, with substantial geographic variability [1, 2]. In current classification systems, including the World Health Organization (WHO) and International Consensus Classification (ICC), EMZL is recognized as a distinct entity alongside splenic and nodal MZL, defined by its extranodal tropism, characteristic morphology, and immunophenotype [3].

EMZL lymphoma is a paradigmatic example of antigen‐driven lymphomagenesis. Chronic immune stimulation—whether from persistent infection or autoimmunity—triggers continuous B‐cell receptor (BCR) engagement, supported by cognate CD4^+^ T‐cell help, cytokines such as BAFF, and formation of organized ectopic lymphoid tissue. This microenvironmental dependence not only underlies the typically indolent clinical course but also explains the remarkable responsiveness of many cases to interventions that remove the inciting stimulus, such as pathogen eradication in gastric or ocular disease [4, 5].

At the molecular level, constitutive NF‐κB activation bridges extrinsic antigenic signals to intrinsic survival pathways. Genetic events, including t(11;18)(q21;q21)/API2–MALT1, and mutations in *TNFAIP3, MYD88*, or *CARD11* progressively uncouple neoplastic B cells from microenvironmental dependence, driving the transition to genetically autonomous disease [6, 7]. These alterations not only illuminate fundamental pathogenetic mechanisms but also carry therapeutic and prognostic significance.

EMZL arises across a spectrum of organs, most commonly the stomach, followed by ocular adnexa, salivary glands, thyroid, lung, and skin. Despite this anatomical heterogeneity, the disease exhibits a conserved histologic signature: small B‐cell infiltrates with monocytoid or centrocyte‐like morphology, lymphoepithelial lesions, and often plasmacytic differentiation. Accurate diagnosis relies on integrating histology, immunophenotype, clonality assessment, and clinical context [8, 9].

Therapeutically, EMZL exemplifies precision medicine: localized antigen‐dependent disease is often controllable—or even cured—with pathogen‐directed antibiotics or limited‐field radiotherapy, whereas systemic or refractory disease necessitates anti‐CD20‐based regimens or novel [10, 11]. Treatment decision, therefore, reflect a synthesis of stage, microenvironmental dependence, genetic lesions, and organ‐specific considerations.

In this review, we provide a comprehensive synthesis of the biological, clinical, and therapeutic perspectives of EMZL. We highlight how understanding into antigenic drive, T‐cell help, and NF‐κB signaling has informed effective, low‐toxicity strategies, while emphasizing ongoing challenges—including histologic transformation, risk stratification, and incomplete dissection of the microenvironmental—that define the cutting edge of research and clinical care.

## 2 Prognosis

EMZL is typically characterized by an indolent clinical course and favorable long‐term survival [12, 13, 14]. Nevertheless, prognostic assessment in this disease has undergone substantial refinement over the past two decades, driven by the recognition that a subset of patients experiences early progression, dissemination, or histologic transformation, thereby adversely impacting survival.

Early prognostic models, including the International Prognostic Index (IPI) for diffuse large B‐cell lymphomas [15], the Follicular Lymphoma International Prognostic Index (FLIPI) [16], and FLIPI2 [17], provided useful frameworks for aggressive and indolent lymphomas, respectively. However, these indices proved insufficiently sensitive for EMZL, failing to capture its distinctive extranodal biology, site‐specific behavior, and typically slow tempo of progression [18, 19]. Several retrospective studies attempted to identify EMZL‐relevant risk factors. In a Korean study of 205 patients with nongastric MZL, nodal MZL subtype, Eastern Cooperative Oncology Group (ECOG) performance status > 1, and advanced‐stage disease (Stages III–IV) independently predicted inferior PFS and OS, leading to the development of a new prognostic index that outperformed both the IPI and the FLIPI [20]. However, the inclusion of nodal MZL and the lack of external validation limited its applicability to EMZL. Similarly, in a cohort of 275 MZL patients—77% with extranodal disease—IPI and FLIPI failed to reliably predict outcome, while elevated serum β2‐microglobulin, male sex, and B symptoms emerged as adverse prognostic factors [21]. Despite their biological plausibility, these early models were not widely adopted in clinical practice.

A major advance came with the development of the EMZL‐IPI [22], derived from 401 patients enrolled in the IELSG‐19 trial [23]. This model incorporates three readily available variables—age > 70 years, Ann Arbor Stage III/IV, and elevated lactate dehydrogenase (LDH) level—and stratifies patients into low, intermediate, and high‐risk groups, with 5‐year event‐free survival (EFS) rates of 70%, 56%, and 29%, respectively. Importantly, the EMZL‐IPI demonstrated prognostic relevance across different treatment modalities, including chlorambucil, rituximab, and their combination, and was externally validated in 633 patients from three independent cohorts. These features established the EMZL‐IPI as the first disease‐specific and clinically robust prognostic tool for EMZL. In the original IELSG‐19 cohort, approximately 42% of patients were classified as low risk, 41% as intermediate risk, and 17% as high risk, underscoring that truly high‐risk EMZL represents only a minority.

More recently, prognostic stratification has been further refined by the Revised EMZL‐IPI, which places PFS at the center of risk assessment—a particularly relevant endpoint in indolent lymphomas [24]. This model incorporates four variables: age > 60 years, elevated LDH, advanced‐stage disease (III–IV), and involvement of multiple extranodal sites [24]. The inclusion of multiple extranodal dissemination represents a key conceptual advance, as it reflects both increased tumor burden and reduced dependence on local microenvironmental support. Compared with the original EMZL‐IPI, the revised score shifts emphasis toward disease‐related characteristics rather than age alone, enabling earlier identification of biologically aggressive disease. Importantly, the Revised EMZL‐IPI, validated across three independent European and US cohorts, stratifies patients into four risk categories and effectively identifies individuals at risk of early progression (POD24), a subgroup associated with significantly inferior lymphoma‐specific survival [24, 25]. This granularity enhances its value for both clinical decision‐making and trial design. In the Revised MALT‐IPI development and validation cohorts, roughly [40%] of patients were assigned to the low‐risk group [34%] and [21%] to the two intermediate categories, and only [5%] to the high‐risk group, again emphasizing that biologically adverse EMZL comprises a small but clinically relevant subset.

Importantly, the clinical relevance of prognostic stratification should be interpreted within the context of the overall favorable natural history of EMZL. Across contemporary cohorts, most patients fall into low‐ or intermediate‐risk categories, while the highest‐risk groups account for only a minority of cases. Nevertheless, these patients contribute disproportionately to early progression events, treatment failure, histologic transformation, and lymphoma‐related mortality. Thus, the primary value of prognostic models may not lie in identifying patients with excellent outcomes—who represent the majority—but rather in recognizing the relatively small subset requiring closer surveillance, biologically informed risk assessment, or earlier systemic intervention.

Despite these advances, prognostic heterogeneity persists. Differences in treatment strategies, baseline characteristics, and geographic biology introduce variability across cohorts. Notably, regional pathogen associations—such as *Chlamydia psittaci* –related ocular adnexal EMZL in Europe but not in the United States—underscore the importance of geographic context in interpreting prognostic models [12, 13, 14]. Prospective validation within contemporary clinical trials and deeper biological characterization of aggressive phenotypes, particularly those associated with multifocal extranodal disease, remain a critical unmet need.

Parallel efforts to establish a unified prognostic framework across all MZL subtypes led to the development of Marginal Zone Lymphoma International Prognostic Index (MZL‐IPI) [26]. Derived from the large NF10 observational cohort and validated in independent US series, this model integrates patients with extranodal, splenic, nodal, and disseminated MZL. Five adverse prognostic factors—elevated LDH, anemia, thrombocytopenia, lymphopenia, and MZL subtype—stratify patients into three risk groups with 5‐year PFS rates of 85%, 66%, and 37%, respectively [26]. While not intended to guide individual treatment decisions in EMZL, the MZL‐IPI provides a valuable framework for clinical trial stratification and cross‐study comparisons, particularly as modern trials increasingly include patients across MZL entities [12, 13, 14, 27].

Beyond tumor‐related factors, patient‐related and social determinants also influence outcome. A large Korean population‐based study demonstrated that comorbid conditions—including diabetes, cardiovascular disease, and cerebrovascular disease—independently worsen survival in EMZL [28]. Conversely, higher socioeconomic status, regular moderate physical activity, nonsmoking status, low to moderate alcohol intake, and higher body mass index were each associated with improved outcomes. These findings underscore the importance of a multidimensional prognostic perspective that integrates biological, clinical, and lifestyle factors alongside established indices such as the EMZL‐IPI.

These observations are particularly relevant when interpreting survival endpoints in EMZL. In population‐based analyses, disease‐specific mortality remains low, whereas deaths from competing causes frequently exceed lymphoma‐related deaths, especially among older patients and those with significant comorbidity burdens. Consequently, deterioration in overall survival often reflects age, cardiovascular disease, diabetes, or other nonlymphoma conditions rather than failure of lymphoma control. This distinction highlights the importance of differentiating disease‐specific outcomes from all‐cause mortality and reinforces the need for prognostic models that identify the uncommon patients at genuine risk of lymphoma‐related death.

Additional refinement has been achieved through incorporation of biological markers. In a multicenter cohort of 468 patients with EMZL treated with contemporary regimens, monoclonal protein (M‐protein) status emerged as a powerful discriminator within the high‐risk EMZL‐IPI category [29]. The resulting extranodal MZL–Monoclonal Protein Index (MZL‐MPI) identified a small subset (approximately 15%) with significantly inferior PFS and OS, while reclassifying a substantial proportion of high EMZL‐IPI patients as low risk. Notably, prognostic discrimination was most evident in patients treated with rituximab monotherapy, highlighting the context‐dependent value of prognostic biomarkers.

Consistent findings have been reported in site‐specific cohorts. In a recent single‐center retrospective analysis at the National Cancer Centre Singapore, including 95 patients with ocular adnexal MZL diagnosed between 1998 and 2024, outcomes were excellent, with 5‐year OS and PFS rates of 94.9% and 84.1%, respectively [30]. Advanced age, higher Ann Arbor stage, and high EMZL‐IPI scores were confirmed as adverse prognostic factors. Exploratory analyses suggested that higher baseline SUVmax on ^18^F‐FDG PET/CT correlated with more advanced disease, raising the possibility that metabolic imaging may contribute to future risk stratification, although independent prognostic significance remains unproven.

Taken together, these data support the preferential use of EMZL‐specific prognostic tools in clinical practice. Among currently available models, the Revised EMZL‐IPI offers the most clinically relevant framework for EMZL, as it was developed and validated in extranodal cohorts, emphasizes PFS, and captures biologically adverse features such as multifocal dissemination [24]. While broader indices retain value for research and trial design, the Revised EMZL‐IPI currently represents the most refined disease‐adapted approach to prognostic assessment in extranodal MZL.

**FIGURE 1:**
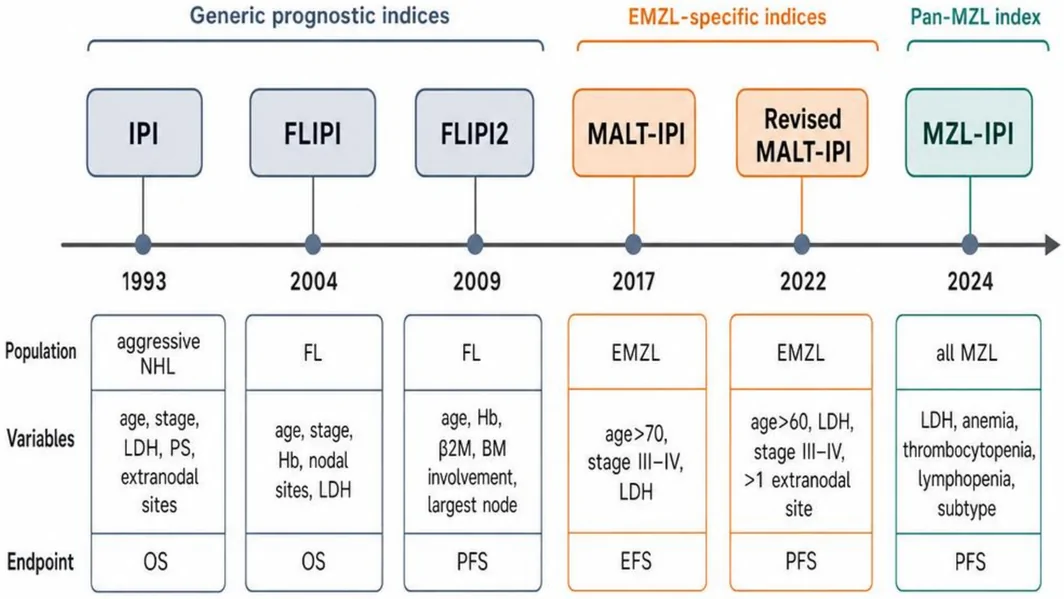
Evolution and scope of prognostic indices relevant to extranodal marginal zone lymphoma (EMZL). Schematic timeline of major lymphoma prognostic indices from 1993 to 2024. For each score (IPI, FLIPI, FLIPI2, MALT‐IPI, Revised MALT‐IPI, MZL‐IPI), the figure summarizes: (i) the primary population for which it was developed (aggressive non‐Hodgkin lymphoma, follicular lymphoma, EMZL, or all marginal zone lymphomas), (ii) the core variables included (e.g., age, Ann Arbor stage, LDH, performance status, number of extranodal sites, cytopenias), and (iii) the principal endpoint (overall survival, event‐free survival, or progression‐free survival). Generic indices (IPI, FLIPI, FLIPI2) are distinguished from EMZL‐specific scores (MALT‐IPI, Revised MALT‐IPI) and the pan‐marginal zone MZL‐IPI, highlighting that only MALT‐IPI and Revised MALT‐IPI were developed and validated in EMZL cohorts, whereas MZL‐IPI integrates all marginal zone subtypes and is primarily intended for cross‐subtype comparisons and trial stratification.

Figure 1 illustrates the conceptual evolution from generic lymphoma prognostic indices to EMZL‐specific and distribution‐aware models, highlighting the increasing emphasis on disease extent, biologic aggressiveness, and early progression risk in EMZL.

## 3 Pathogenesis

EMZL represents a paradigmatic model of lymphomagenesis driven by interplay between chronic antigenic stimulation, sustained microenvironmental support, and the stepwise acquisition of genetic lesions that converge on constitutive activation of NF‐κB signaling [4, 5]. Unlike most indolent B‐cell neoplasms, EMZL often remains partially dependent on its etiologic trigger and local immune context, a feature that defines both its unique biology and its distinctive therapeutic vulnerabilities [31].

### 3.1 Chronic Antigenic Drive and Ectopic Lymphoid Tissue Formation

The initiating event in EMZL lymphomagenesis is the development of acquired lymphoid tissue at extranodal sites that normally lack organized lymphoid structures. Persistent antigenic stimulation—most convincingly demonstrated for *Helicobacter pylori* infection in the stomach—induces chronic inflammation, recruitment of T cells, antigen‐presenting cells, and B cells, and the formation of ectopic germinal center–like structures. While this process recapitulates physiological mucosa‐associated immune responses, prolonged antigenic exposure transforms it into a pathological niche that supports malignant evolution [32, 33].

The causal relationship between *H. pylori* and gastric EMZL is firmly established by epidemiologic and experimental evidence, as well as by the consistent observation that bacterial eradication induces lymphoma regression in a substantial proportion of early‐stage cases [1, 34]. Analogous, though less universal, associations have been described in selected nongastric sites, including *Chlamydophila psittaci* in ocular adnexal EMZL [35], *Borrelia burgdorferi* in subsets of cutaneous cases [36], and *Campylobacter jejuni* in immunoproliferative small intestinal disease [1, 37, 38]. In addition, autoimmune disorders such as Sjögren syndrome or Hashimoto thyroiditis promote sustained immune activation and lymphoid accumulation, further reinforcing antigen‐driven lymphomagenesis [39].

A key mechanistic link between chronic immune stimulation and genetic evolution was provided by the demonstration that EMZL is targeted by aberrant somatic hypermutation (aSHM), affecting both immunoglobulin genes and proto‐oncogenes such as *PIM1, PAX5*, and *MYC* [40]. These findings established EMZL lymphoma as a neoplasm shaped by deregulated physiological DNA editing within ectopic germinal center–like structures. The presence of similar aSHM patterns in indolent EMZL and in DLBCL transformed from EMZL supports a model of gradual genetic evolution rather than abrupt transformation. Clinically, this paradigm explains how infection‐driven EMZL may persist for years while retaining a measurable risk of late progression.

### 3.2 T‐Cell Help and Microenvironmental Dependence

Dependence on the local immune microenvironment is a defining feature of early EMZL, with CD4^+^ T‐cell help playing a central pathogenic role. In infection‐driven settings such as *H. pylori* –associated gastric disease, tumor‐infiltrating T cells recognize microbial antigens and provide cognate help to neoplastic B cells through CD40–CD40L interactions, costimulatory signals, and Th2‐skewed cytokines, including interleukin‐4 and interleukin‐10 [41].

This T‐cell–dependent support is reinforced by additional microenvironmental components. BAFF, produced by myeloid cells, stromal elements, and occasionally the lymphoma cells themselves, delivers potent survival signals to marginal zone–type B cells and amplifies NF‐κB activation [42]. Follicular dendritic cells and other antigen‐presenting cells maintain chronic antigen exposure and tissue architecture, while stromal and endothelial components shape chemokine gradients and adhesion signals that retain lymphoma cells within these supportive niches. Uhl et al. provide a detailed overview of how chronic inflammation recruits and activates immune and stromal elements in EMZL, fostering a supportive milieu for malignant B cells [43, 44, 45].

Recent high‐resolution studies now provide direct cellular confirmation of this microenvironmental dependence. Single‐cell RNA‐sequencing analysis of ocular adnexal EMZL revealed a malignant compartment composed almost exclusively of clonal memory B cells embedded within a highly structured immune niche characterized by active CD70–CD27 and CXCL13–CXCR5 signaling and a bias of CD4^+^ naïve T cells toward T‐follicular helper differentiation [45]. In contrast, IgG4‐related ophthalmic disease displays a nonneoplastic, germinal‐center–skewed B‐cell population within a regulatory Treg‐enriched microenvironment, offering a mechanistic explanation for the morphologic and clinical overlap between these entities.

Importantly, the composition of the supportive microenvironment varies according to the anatomic site of origin. Gastric EMZL lymphomas are typically enriched in *H. pylori* –reactive T cells, macrophages, and BAFF‐producing inflammatory cells, reflecting their dependence on chronic bacterial stimulation. In contrast, salivary gland and thyroid EMZL lymphomas arising in Sjögren syndrome or Hashimoto thyroiditis develop within autoimmune niches characterized by abundant T‐helper cells, plasma cells, and ectopic germinal centers. Ocular adnexal lesions appear to rely more heavily on follicular helper T‐cell programs, CXCL13‐mediated cell trafficking, and organized immune‐cell interactions. These observations suggest that, although NF‐κB activation represents a common downstream pathway, the upstream microenvironmental networks supporting lymphoma growth are partially site‐specific.

This reliance on antigen‐specific T‐cell help provides a biological explanation for the indolent behavior and therapeutic reversibility of early EMZL. Disruption of the inciting antigen—most notably through *H. pylori* or *C. psittaci* eradication—can dismantle T‐cell help and cytokine support, leading to durable tumor regression in a substantial proportion of patients [33, 35]. With disease progression, however, accumulating genetic lesions progressively reduce reliance on these external signals, marking the transition toward microenvironment‐independent growth.

### 3.3 Genetic Lesions and Activation of NF‐KB and Notch Pathways

A defining hallmark of EMZL is dysregulation of the NF‐κB signaling network mediated by recurrent genetic lesions that confer survival and proliferative advantages. The most characteristic alterations are chromosomal translocations that directly engage NF‐κB signaling. The t(11;18)(q21;q21) API2–EMZL1 fusion results in constitutive EMZL1 activation of both canonical and noncanonical NF‐κB pathways and is strongly associated with resistance to *H. pylori* eradication, disseminated disease and reduced microenvironmental dependence [46]. Additional translocations involving *BCL10* and *EMZL1* with *IGH* similarly converge on NF‐κB activation [4, 46].

Beyond translocations, inactivation of negative regulators further contributes to pathway dysregulation. Loss‐of‐function mutations or deletions affecting *TNFAIP3* (A20) abrogate its inhibitory control over canonical NF‐κB signaling and are recurrent in EMZL, particularly, at sites such as the ocular adnexa and thyroid [4]. Alterations in other NF‐κB regulators, including *TRAF3* or *NFKBIA*, occur in a subset of translocation‐negative cases, highlighting the diversity of mechanisms leading to pathway activation [47].

In contrast to other B‐cell lymphomas, activating mutations in proximal BCR‐associated scaffolds such as *CARD11* are rare in typical extranodal disease, underscoring a genetic landscape distinct from that of DLBCL and some nodal/splenic MZLs [48].

Recurrent mutations affecting the NOTCH signaling pathway, particularly *NOTCH1* and *NOTCH2*, have been identified in subsets of EMZL—most notably in ocular adnexal disease—suggesting that NOTCH dysregulation may cooperate with NF‐κB‐driven survival signals in disease progression [49, 50].

Emerging genomic studies also indicate that the distribution of genetic lesions is not uniform across anatomical sites. Gastric EMZL are enriched for t(11;18)(q21;q21)/API2–EMZL1, a lesion associated with resistance to *H. pylori* eradication and reduced dependence on antigenic stimulation. Ocular adnexal lymphomas more frequently harbor alterations involving TNFAIP3, NOTCH1/2, and other regulators of NF‐κB signaling, whereas salivary gland and thyroid EMZL lymphomas often arise in the context of autoimmune disease and display a broader spectrum of mutations affecting immune‐regulatory pathways. These site‐specific genetic patterns likely reflect distinct selective pressures imposed by local inflammatory milieus and may contribute to differences in clinical behavior, therapeutic responsiveness, and risk of dissemination.

Collectively, EMZL exemplifies a continuum from antigen‐dependent proliferation to genetically autonomous growth. Early disease—most notably *H. pylori* ‐driven gastric EMZL—relies on chronic immune stimulation and microenvironmental support, a dependency reflected clinically by frequent regression following pathogen eradication.

Disease progression is driven by the accumulation of genetic lesions that activate NF‐κB independently of antigenic input, conferring resistance to pathogen‐directed therapy, promoting dissemination, and enabling occasional transformation to aggressive lymphoma. This dependency spectrum provides a unifying framework linking pathogenesis to prognosis and therapy, and underscores the need for precision‐guided risk stratification and targeted interventions.

**FIGURE 2:**
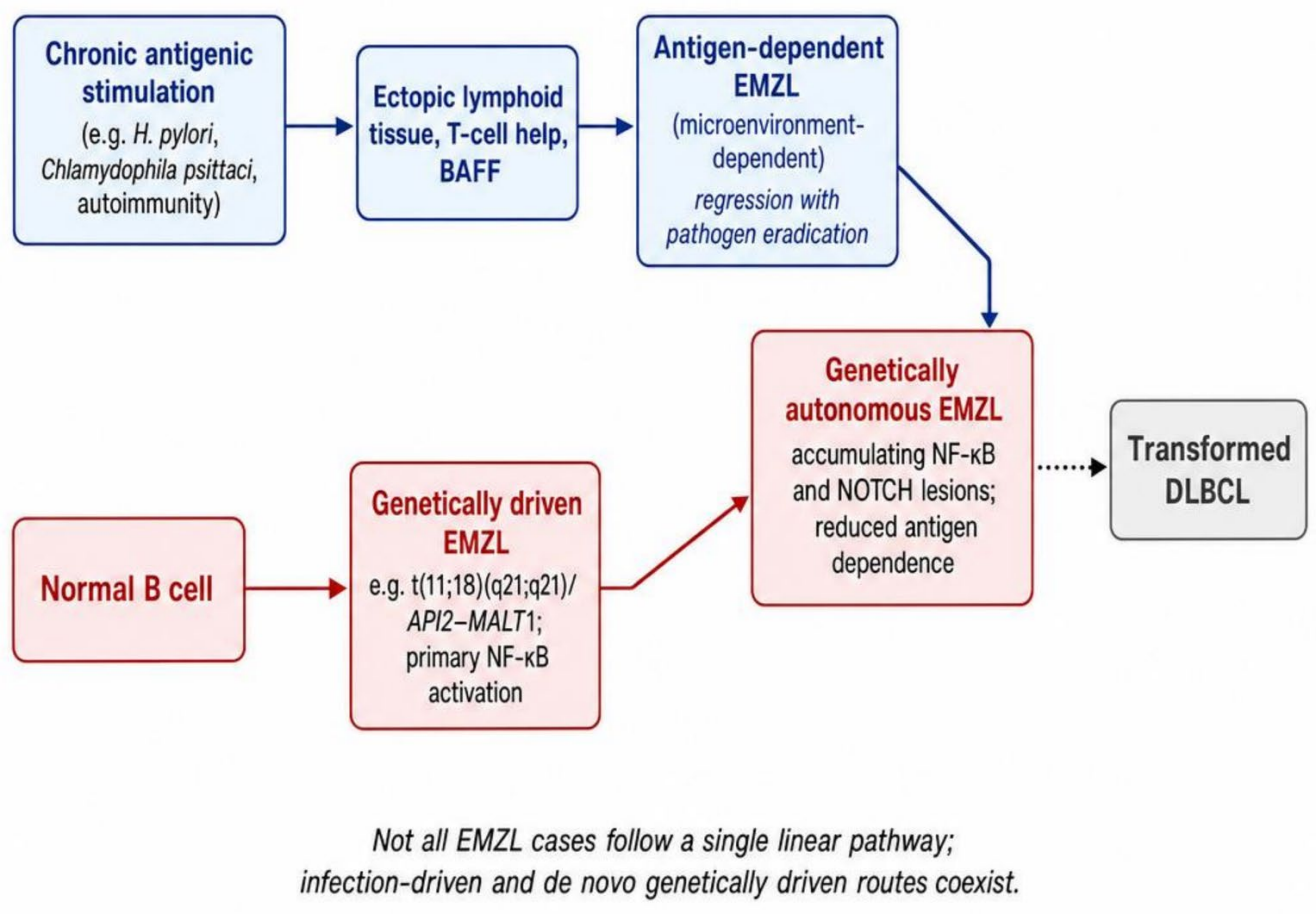
Branched pathogenetic model of EMZL: From antigen dependence to genetic autonomy. Conceptual schema illustrating the biological continuum of EMZL while accounting for distinct pathogenetic routes. On the left branch, chronic antigenic stimulation (e.g., *Helicobacter pylori* , *Chlamydophila psittaci* , autoimmune disorders) induces ectopic lymphoid tissue formation, T‐cell help, BAFF signaling, and a supportive microenvironment, leading to antigen‐dependent, microenvironment‐dependent EMZL that may regress after pathogen eradication. On the right branch, normal B cells acquire primary genetic lesions such as t(11;18)(q21;q21)/API2–MALT1 and other NF‐κB‐activating events, giving rise to relatively antigen‐independent EMZL from the outset. Both branches converge on genetically autonomous EMZL characterized by accumulating NF‐κB and NOTCH pathway lesions and reduced reliance on the microenvironment. A dotted arrow indicates the potential, albeit infrequent, evolution to transformed diffuse large B‐cell lymphoma. The figure emphasizes that EMZL does not follow a single linear progression and that de novo genetically driven cases coexist with infection‐driven, microenvironment‐dependent disease.

The transition from antigen‐dependent to genetically autonomous lymphoma is schematically illustrated in Figure 2.

## 4 Clinical Presentation and Diagnostic Evaluation

EMZL arises in organs that either harbor native EMZL—most notably the gastrointestinal tract—or acquire lymphoid tissue as a consequence of chronic antigenic stimulation. Prototypical examples include *H. pylori* –associated gastritis, autoimmune thyroiditis in Hashimoto disease, and chronic sialadenitis in Sjögren syndrome [2, 51]. In this context, the diagnosis hinges on careful integration of morphology, immunophenotype, molecular clonality, and clinical setting, as the distinction between lymphoma and reactive lymphoid proliferations is often subtle.

Across anatomic sites, EMZL displays a broadly conserved histologic appearance. The infiltrate is typically composed of small B cells with centrocyte‐like or monocytoid morphology, accompanied by variable plasmacytic differentiation, colonization of reactive follicles, and lymphoepithelial lesions. Immunophenotypically, the neoplastic cells express CD20, CD79a, and PAX5, frequently show surface IgM, and are usually negative for CD5, CD10, and cyclin D1. Demonstration of light‐chain restriction can support clonality but must always be interpreted in the context of cytologic features, particularly in tissues affected by chronic inflammation [2, 8].

Despite shared features, diagnostic interpretation is highly organ‐dependent, and pitfalls arise from overlap with site‐specific reactive or inflammatory conditions. In the gastrointestinal tract, particularly, the stomach and small intestine, early EMZL may closely resemble chronic active gastritis or enteritis with prominent lymphoid follicles and reactive marginal zones. Architectural distortion, confluent lymphoepithelial lesions, and reproducible evidence of clonality (by light‐chain restriction or IGH rearrangement) favor lymphoma over reactive conditions [2, 8] but may be focal or absent in early disease.

Similar challenges are encountered in salivary glands and thyroid, where dense lymphoid infiltrates with germinal centers are common in autoimmune sialadenitis or thyroiditis. Overdiagnosis may occur when reactive lymphoid hyperplasia is misinterpreted as neoplasia, while underdiagnosis is possible when malignant infiltrates are patchy or dominated by plasmacytic differentiation. In such settings, correlation with clinical findings and judicious use of clonality studies are essential [2, 8].

Ocular adnexal EMZL presents a particularly demanding diagnostic scenario due to its close morphologic and immunophenotypic overlap with reactive IgG4‐related ophthalmic disease. Assessment of IgG4‐positive plasma cell density, fibrosis patterns, and obliterative phlebitis is therefore integral to routine practice [8]. Recent single‐cell RNA‐sequencing data have provided mechanistic insight into this distinction, demonstrating that ocular adnexal EMZL is composed of clonal malignant memory B cells embedded in a structured immune niche, whereas IgG4‐related ophthalmic disease shows a nonneoplastic, germinal‐center–skewed B‐cell population within a regulatory, plasma cell–rich microenvironment [45]. The overlap between these entities likely reflects a common background of chronic antigen‐driven immune activation. Both lesions are characterized by lymphoid follicle formation, plasma‐cell infiltration, and cytokine‐mediated tissue remodeling, which can generate similar architectural and histologic patterns. However, whereas IgG4‐related disease is sustained by a polyclonal immune response enriched in regulatory T cells, plasmablasts, and profibrotic cytokines such as IL‐10 and TGF‐β, ocular adnexal EMZL is driven by the emergence of a clonal memory B‐cell population that progressively acquires growth and survival advantages while remaining embedded within an antigen‐responsive microenvironment. These differences explain why morphologic similarities may coexist with profoundly distinct biological behavior. While informative, these molecular findings complement rather than replace conventional histopathologic evaluation.

Comparable diagnostic challenges arise at other extranodal sites, including the skin and lung, where lymphoid infiltrates may mimic cutaneous pseudolymphoma or follicular bronchiolitis, respectively. In these settings, accurate classification requires integration of histopathology with radiologic findings, clinical course, and molecular studies [51, 52].

Accurate staging is essential to guide management and typically includes imaging of involved regions and assessment for dissemination. While ^18^F‐FDG PET/CT may provide useful information for nongastric or bulky lesions, its sensitivity in low‐grade EMZL, particularly gastric disease, is limited. Consequently, contrast‐enhanced CT or MRI remains the standard imaging modality in most clinical scenarios, with bone marrow evaluation reserved for selected cases [2, 3].

Overall, diagnosis of EMZL requires an integrated approach that synthesizes morphology, immunophenotype, molecular findings, and site‐specific clinical context. The distinction between reactive and neoplastic lymphoid proliferations is often subtle, especially in chronically inflamed tissues. In equivocal cases, descriptive diagnoses such as “atypical lymphoid proliferation” with close clinical correlation and follow‐up are appropriate. This cautious, multidisciplinary strategy minimizes both overtreatment and underrecognition, ensuring accurate classification of this biologically and clinically distinctive lymphoma [2, 51, 52].

**FIGURE 3:**
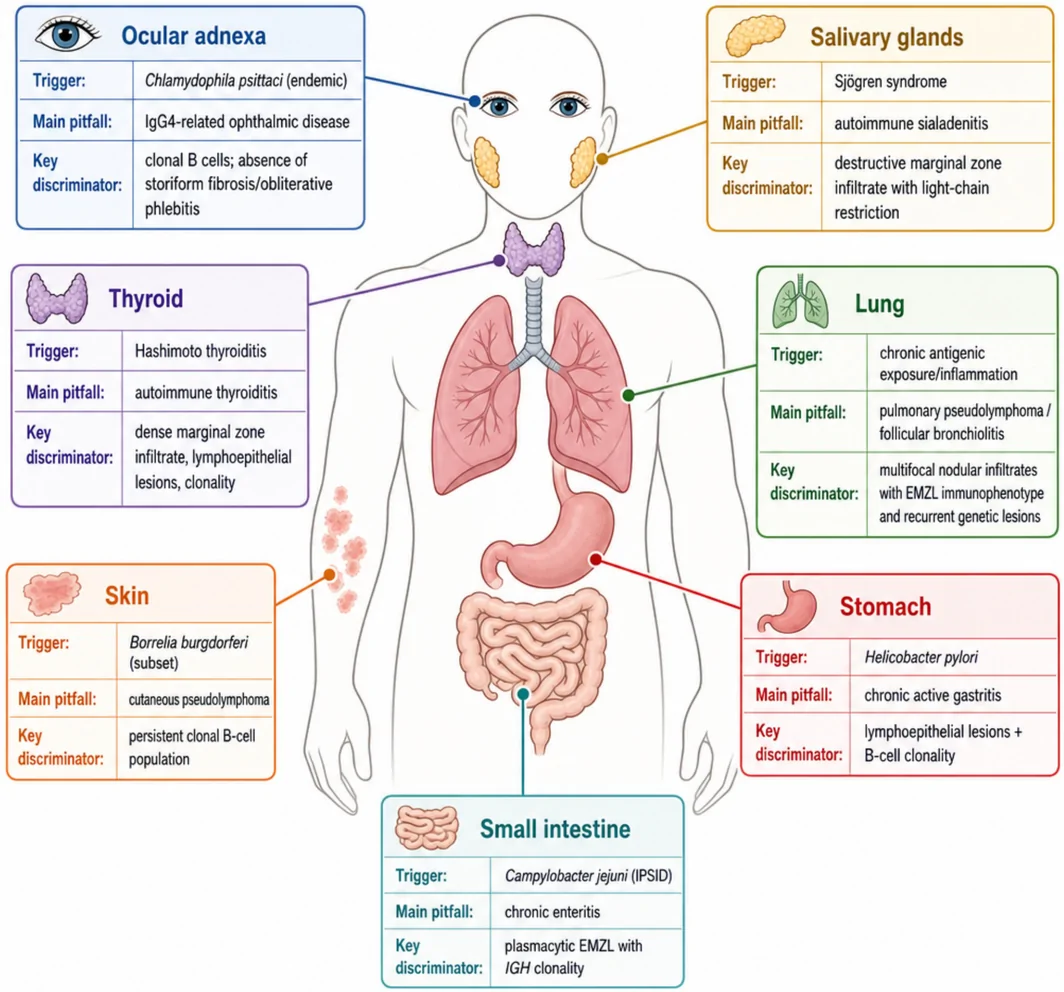
Site‐specific presentations and diagnostic pitfalls in EMZL. Schematic representation of the principal anatomic sites involved by EMZL, with organ‐specific callouts summarizing three key elements: (i) predominant etiologic trigger or association, (ii) main reactive or inflammatory condition that can mimic EMZL, and (iii) critical discriminator favoring lymphoma. For gastric EMZL, *Helicobacter pylori* –associated chronic gastritis is the main trigger and pitfall, with lymphoepithelial lesions and evidence of B‐cell clonality supporting diagnosis. In the ocular adnexa, *Chlamydophila psittaci* infection (in endemic regions) and IgG4‐related ophthalmic disease represent the typical association and pitfall; clonal B cells and absence of classic IgG4‐related features (storiform fibrosis, obliterative phlebitis) favor EMZL. Salivary gland and thyroid EMZL arise in the context of Sjögren syndrome and Hashimoto thyroiditis, respectively, and must be distinguished from autoimmune sialadenitis/thyroiditis based on destructive marginal zone infiltrates, lymphoepithelial lesions, and clonality. Pulmonary EMZL may be confused with pseudolymphoma or follicular bronchiolitis, while primary cutaneous EMZL can mimic cutaneous pseudolymphoma; in both settings, persistent, multifocal infiltrates with a characteristic marginal zone immunophenotype and reproducible clonal rearrangements support neoplasia. The figure underscores the need for integrated clinical, histologic, immunophenotypic, and molecular assessment to avoid both over‐ and under‐diagnosis.

Figure 3 summarizes the site‐specific presentations and diagnostic pitfalls of EMZL, highlighting the overlap with reactive and inflammatory conditions and reinforcing the necessity of an integrated diagnostic approach.

## 5 Therapy of EMZL

EMZL is unique among indolent B‐cell neoplasms in that, in a substantial subset of cases, the driving antigenic stimulus and its associated microenvironment remain therapeutically exploitable. As a result, the therapeutic strategy must be tailored not only to disease stage and patient fitness but also to the underlying etiology, with a consistent preference for the least intensive intervention that provides durable disease control.

### 5.1 Antibiotic Therapy in Gastric and Ocular EMZL

In localized gastric EMZL associated with *H. pylori* , eradication of the infection is the cornerstone of initial management. The seminal observation by Wotherspoon and colleagues [33] that low‐grade gastric EMZL can regress completely after *H. pylori* eradication has been confirmed in multiple large series, demonstrating complete histologic remission in approximately 60%–80% of early stage, t(11;18)‐negative cases, with excellent 5‐ and 10‐year overall survival and very low rates of systemic progression or transformation [34, 53, 54, 55, 56].

In this setting, antibiotics alone are a rational and definitive first‐line therapy. Endoscopic and histologic surveillance is mandatory, as responses may be delayed for many months, and additional local or systemic treatment should be deferred unless there is clear evidence of persistence or progression after an adequate observation period. By contrast, gastric EMZL that are *H. pylori–*independent at diagnosis, refractory to eradication, or harbor the t(11;18)(q21;q21)/API2–EMZL1 translocation rarely regress with antibiotics and typically require radiotherapy or systemic immunochemotherapy.

A similar etiologic and therapeutic paradigm applies, albeit in a more restricted and geographically variable fashion, to selected nongastric EMZL. In ocular adnexal MZLs from regions endemic for *C. psittaci* , pathogen‐directed antibiotic therapy can induce meaningful responses. In an international phase II trial of patients with stage I ocular adnexal EMZL, first‐line doxycycline achieved an overall response rate of approximately 65%, with 5‐year PFS of ~55%; responses were more frequent and durable when microbiologic eradication was documented [57].

Across both gastric and nongastric disease, the guiding principle is respect for the indolent biology of EMZL. Systemic treatment should be avoided unless clearly indicated by multifocal or advanced‐stage disease, symptomatic or organ‐threatening involvement, or failure of eradication or local therapy. Collectively, these data establish pathogen‐directed antibiotics as a low‐toxicity, mechanism‐based, and often highly effective first‐line strategy in infection‐driven EMZL, with escalation reserved for biologically autonomous or disseminated cases.

### 5.2 Radiotherapy as Definitive Local Treatment

Involved‐site radiotherapy remains a highly effective and often curative option for stage I–II EMZL at many nongastric sites, including the ocular adnexal, salivary gland, thyroid, lung, and skin. Moderate‐dose radiation dose (typically 24–30 Gy) achieves excellent local control and durable progression‐free survival [58, 59, 60].

Large single‐institution and multicenter cohorts, including an international series of ocular adnexal EMZL, report 5‐year local control and PFS rates of 80%–90%, with acceptable late toxicity when modern radiotherapy techniques are employed [58, 59]. In clinical practice, radiotherapy is therefore preferred for localized, symptomatic disease in anatomically suitable regions when eradication or observation is not appropriate. Systemic therapy is reserved for multifocal, advanced‐stage, or relapsed disease.

### 5.3 Anti‐CD20‐Based Systemic Therapy in First Line

When systemic treatment is required—because of disseminated involvement, high tumor burden, organ‐threatening disease, or failure of local and eradication strategies—anti‐CD20‐based regimens form the therapeutic backbone. The randomized IELSG‐19 trial established rituximab plus chlorambucil as a reference combination, demonstrating higher complete response rates and significantly prolonged event‐free and progression‐free survival compared with either agent alone, while overall survival remained excellent across all arms [23].

For fit patients with higher‐risk or advanced‐stage disease, bendamustine–rituximab (BR) is highly active. BR induced complete or unconfirmed complete responses in nearly all patients and achieved a 7‐year progression‐free survival exceeding 90% [61]. An international retrospective analysis of 237 front‐line BR‐treated patients confirmed its efficacy but also highlighted increased infectious risk and late toxicity in unselected, often older populations. These data support a risk‐adapted approach, reserving BR for younger, fit patients with clear indications for systemic therapy, while favoring rituximab–chlorambucil or rituximab monotherapy in older or comorbid individuals [23].

### 5.4 Targeted Agents and Chemo‐Free Combinations in Relapsed/Refractory Disease

For patients with relapsed or refractory EMZL after prior anti‐CD20‐based therapy, targeted agents and chemo‐free combinations have expanded the therapeutic landscape. The first‐in‐class BTK inhibitor ibrutinib demonstrated durable activity in relapsed/refractory MZL, including EMZL cases, with overall response rates of approximately 50%–60% and median progression‐free survival approaching 2 years [62]. The next‐generation BTK inhibitor zanubrutinib confirmed these findings in the MAGNOLIA study, showing high response rates, durable disease control, and a favorable safety profile [63].

The immunomodulatory combination lenalidomide–rituximab has also shown activity in indolent lymphomas. In the Phase III AUGMENT trial, this combination significantly improved response rates and PFS compared with rituximab alone, with exploratory analyses suggesting similar benefit in MZL and follicular lymphoma [64]. Although these agents are unlikely to replace local or antibody‐based approaches in first‐line EMZL, they represent important options in later lines, particularly for chemorefractory patients or those in whom further alkylator exposure is undesirable.

### 5.5 CAR T‐Cell Therapy in EMZL

CD19‐directed CAR T‐cell therapy is becoming a credible option for relapsed/refractory EMZL within the broader MZL spectrum, particularly for patients who have exhausted standard immunochemotherapy and targeted approaches. In the global, multicohort, single‐arm Phase 2 TRANSCEND FL study, Palomba et al. reported primary analysis results for lisocabtagene maraleucel (liso‐cel) in r/r MZL, a clinically relevant population because it reflects real‐world complexity with heavy pretreatment (median 3 prior lines) and includes an EMZL subgroup (17/66 efficacy‐evaluable patients). Efficacy was notable, with an overall response rate of 95% (63/66) and complete responses in 80% (53/66), suggesting that deep remissions are achievable even in indolent B‐cell disease traditionally managed with sequential noncurative therapies. At a median follow‐up of 24.1 months, the median duration of response, progression‐free survival, and overall survival were not reached, supporting the possibility of durable disease control after a single cellular infusion in at least a proportion of patients. From a safety standpoint—critical when considering CAR T in indolent lymphomas and older/frail patients—high‐grade immune effector toxicities were uncommon: Grade 3 cytokine release syndrome occurred in 4% (3/67) and Grade 3 neurologic events in 4% (3/67), with no Grades 4–5 CRS or neurologic events reported; grade ≥ 3 infections occurred in 16% (11/67), emphasizing the need for robust infectious prophylaxis/monitoring strategies but supporting overall feasibility. Consistent with evolving lymphoma guidance that increasingly incorporates immune effector cell therapy for appropriate relapsed/refractory B‐cell lymphomas in expert centers, these data position liso‐cel as a promising, potentially practice‐changing option for selected r/r MZL patients, including those with EMZL, where achieving deep, durable remissions is a key unmet need [65].

### 5.6 Response Assessment, Depth of Remission, and Treatment Goals

Assessment of therapeutic success in EMZL is also evolving. Overall survival is a relatively insensitive endpoint in first‐line EMZL, where lymphoma‐related mortality is low and nonlymphoma deaths predominate. Prospective data demonstrate that achieving complete remission within 24 months and the time required to reach that remission are highly predictive of long‐term progression‐free survival, with strong surrogacy at both individual‐patient and trial levels.

Multiple studies have also demonstrated the prognostic value of ^18^F‐FDG PET/CT, with baseline metabolic burden and interim PET response correlating with long‐term outcomes in both gastric and nongastric EMZL, although interpretation must remain site‐specific and complemented by endoscopic and histologic assessment in gastric disease [66, 67, 68, 69]. Emerging data on minimal residual disease and circulating tumor DNA further suggest that molecular response assessment may eventually refine treatment duration and intensity in EMZL [70, 71].

Collectively, these data support a therapeutic philosophy centered on precision and restraint, exploiting residual antigenic and microenvironmental dependence whenever possible, and reserving systemic or targeted therapy for cases demonstrating molecular autonomy, dissemination, or treatment resistance. Early depth of response, durability of disease control, and preservation of quality of life—rather than crude survival endpoints—should define therapeutic success in EMZL.

**FIGURE 4:**
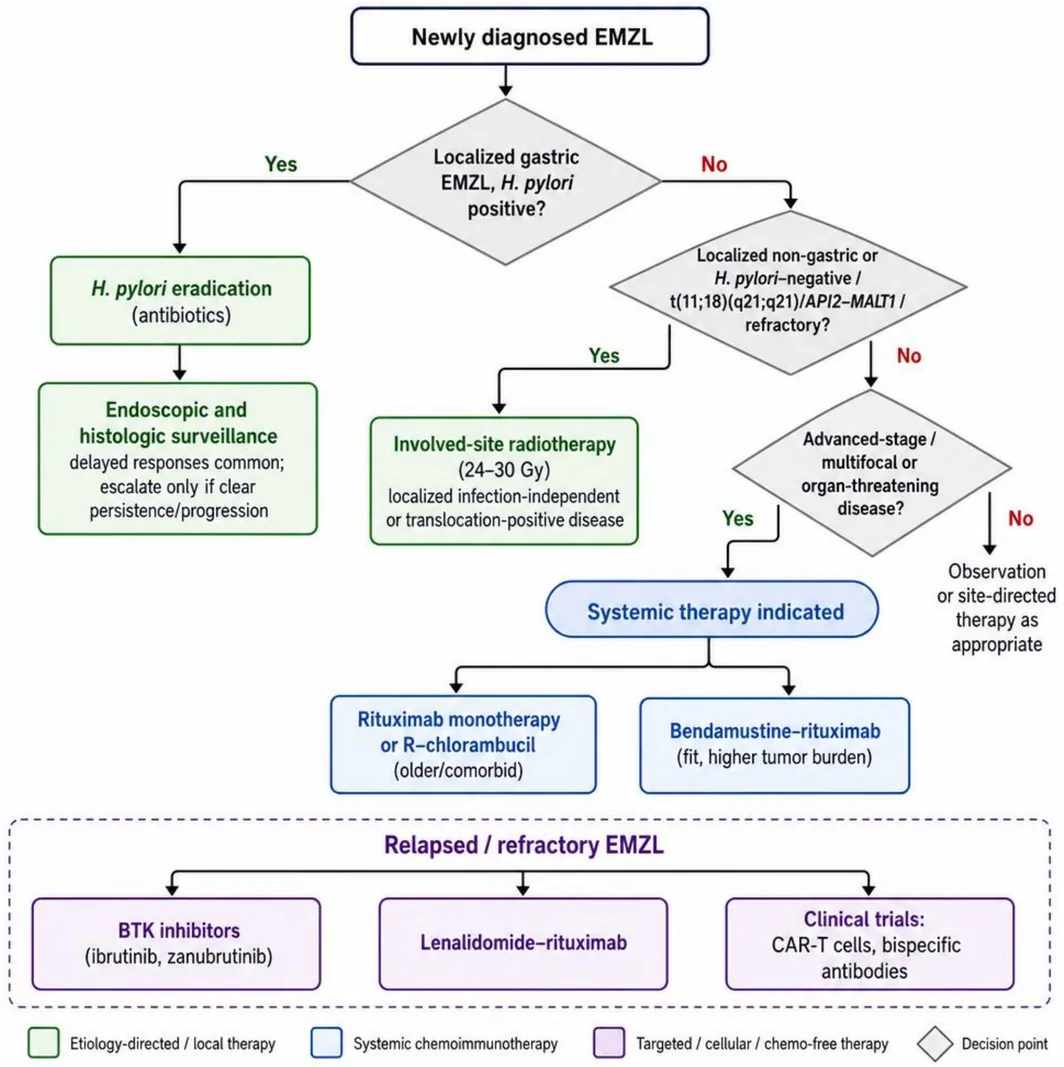
Therapeutic algorithm for extranodal marginal zone lymphoma (EMZL). Biology‐ and site‐adapted treatment flowchart for EMZL. Newly diagnosed EMZL is first stratified by site and etiology. In localized gastric EMZL associated with *Helicobacter pylori* , eradication therapy is the recommended initial approach, followed by endoscopic and histologic surveillance; delayed responses are common, and escalation to radiotherapy or systemic therapy is reserved for clearly persistent or progressive disease. Localized, nongastric EMZL or gastric EMZL that is *H. pylori* –independent, refractory to eradication, or harboring t(11;18)(q21;q21)/API2–MALT1 is typically managed with involved‐site radiotherapy (approximately 24–30 Gy), achieving excellent local control. Advanced‐stage, multifocal, or organ‐threatening EMZL warrants systemic therapy: Options include rituximab monotherapy or rituximab–chlorambucil for older or comorbid patients, and bendamustine–rituximab for fit individuals with higher tumor burden. In the relapsed or refractory setting, targeted and chemo‐free strategies such as Bruton tyrosine kinase inhibitors (e.g., ibrutinib, zanubrutinib) and lenalidomide–rituximab are considered, with enrolment in clinical trials of novel cellular or immunotherapies (e.g., CAR‐T cells, bispecific antibodies) encouraged where available. The algorithm emphasizes minimal effective intervention, de‐escalation when possible, and escalation based on disease extent, biology, and patient fitness.

Figure 4 schematically summarizes this stepwise, biology‐driven treatment algorithm.

## 6 Discussion

EMZL remains a biologically distinctive entity of MZL, and recent integrative analyses have further clarified its unique position within the broader landscape of marginal zone B‐cell neoplasms. These conceptual advances are supported by a growing body of primary molecular, translational, and clinical studies that have progressively refined our understanding of EMZL biology. Throughout this review, emphasis has been placed on landmark investigations that established the roles of chronic antigenic stimulation, NF‐κB pathway activation, and site‐specific genetic alterations, while recent reviews have been used primarily to contextualize and integrate these findings. As emphasized by Laurent and Bertoni, while MZL subtypes share overlapping morphologic and immunophenotypic features, EMZL is defined by its tight linkage to chronic antigenic stimulation, site‐specific genetic alterations, and sustained dependence on the local microenvironment—features that collectively shape its pathogenesis, clinical behavior, and therapeutic vulnerabilities [6].

At the cellular and molecular level, early EMZL lymphomagenesis is driven by persistent antigenic stimuli—most prominently *H. pylori* in the stomach and *C. psittaci* in the ocular adnexa—that promote the formation of ectopic lymphoid tissue and sustain B‐cell activation through T‐cell help, BAFF signaling, and other microenvironmental cues. This antigen‐dependent phase is characterized by a dynamic interplay between neoplastic marginal zone–like B cells and their immune niche, rendering the lymphoma uniquely susceptible to interventions that disrupt the initiating stimulus. With disease evolution, however, the acquisition of recurrent genetic lesions—including t(11;18)(q21;q21)/API2–EMZL1, inactivation of negative regulators such as *TNFAIP3*, and activating mutations in *MYD88* or *CARD11*—leads to constitutive NF‐κB activation, progressively reducing reliance on external antigenic drive and marking a transition toward greater biological autonomy.

This biological continuum is directly mirrored in clinical behavior and therapeutic responsiveness. Pathogen‐directed therapy provides the clearest illustration of immune reversibility in human lymphoma, with *H. pylori* eradication achieving durable complete remissions in a substantial proportion of patients with early‐stage gastric EMZL. In selected geographic settings, antibiotic therapy targeting *C. psittaci* has demonstrated comparable activity in ocular adnexal disease. In localized, nongastric extranodal sites where infectious drivers are less consistent or absent, moderate‐dose involved‐site radiotherapy offers excellent local control and prolonged progression‐free survival. Conversely, disseminated, symptomatic, or refractory disease typically requires systemic treatment, with anti–CD20–based regimens such as rituximab–chlorambucil or bendamustine–rituximab producing high response rates and durable disease control. More recently, chemo‐free approaches—including BTK inhibitors and lenalidomide–rituximab—have expanded therapeutic options in the relapsed or refractory setting [10].

Despite these advances, several unmet clinical and biological needs remain. An additional issue concerns the selection of clinically meaningful endpoints in EMZL. Given the prolonged survival of most patients and the relatively low incidence of lymphoma‐related death, overall survival is often insensitive to biologically relevant differences between treatment strategies. In contrast, progression‐free survival, POD24, time to next treatment, and lymphoma‐specific survival may better capture the consequences of treatment resistance and disease evolution. We therefore believe that, although overall survival remains the most objective endpoint, it should not be considered the sole measure of clinical benefit in EMZL. Future studies should prioritize endpoints capable of identifying the minority of patients who experience early progression, transformation, or lymphoma‐related mortality, while recognizing that many patients will ultimately die with the disease rather than from it. First, there is a critical lack of reliable predictive biomarkers capable of identifying patients who will achieve long‐term disease control with conservative, low‐toxicity strategies versus those who require early systemic therapy. Emerging approaches—such as molecular minimal residual disease assessment, circulating tumor DNA analysis, and advanced imaging metrics—hold promise but remain investigational and require prospective validation before clinical implementation. Second, histologic transformation, while relatively uncommon in EMZL compared with other MZL entities, represents a clinically meaningful event with substantial prognostic impact. Recent prospective and meta‐analytic data indicate transformation rates of approximately 3% at 5 years and 5% at 10 years in extranodal disease, with transformation strongly associated with inferior survival outcomes. Identified risk factors, including multiple extranodal sites and higher baseline risk scores, underscore the need for tools that allow earlier identification of patients at increased risk [65].

A third and closely related challenge lies in the incomplete characterization of the tumor microenvironment. While its central role in antigen dependence and immune escape is well established, the precise contributions of distinct immune cell subsets, stromal elements, and cytokine networks—and how these components evolve over time or under therapeutic pressure—remain incompletely defined. High‐resolution approaches, including single‐cell and spatial profiling, are beginning to elucidate these context‐dependent interactions and may ultimately reveal novel opportunities for immunomodulatory or microenvironment‐directed therapies.

**FIGURE 5:**
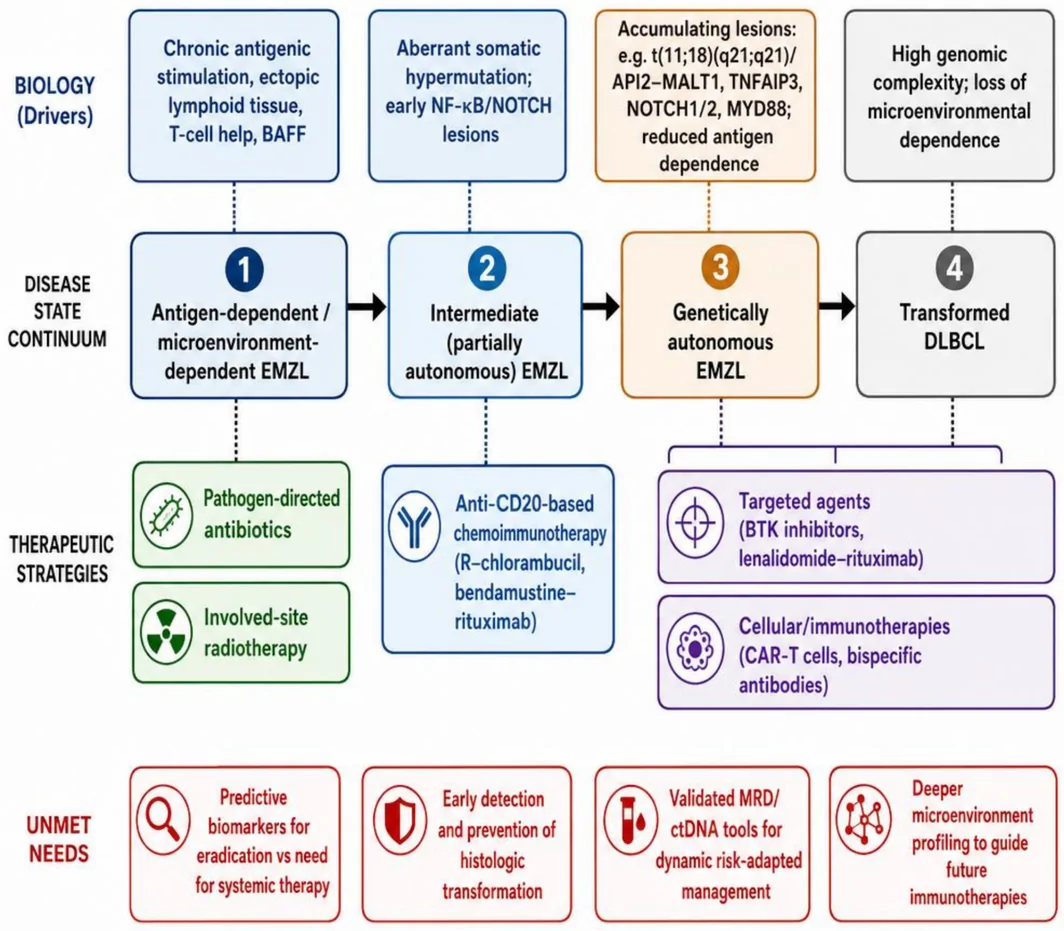
Integrated model of EMZL biology, therapeutic strategies, and unmet clinical needs. Conceptual framework aligning disease biology, treatment approaches, and outstanding challenges along a continuum from antigen‐dependent to genetically autonomous EMZL and transformed lymphoma. The horizontal axis depicts four stages: (1) antigen‐dependent, microenvironment‐dependent EMZL driven by chronic antigenic stimulation, T‐cell help, BAFF, and ectopic lymphoid tissue; (2) intermediate, partially autonomous EMZL shaped by aberrant somatic hypermutation and early NF‐κB/NOTCH pathway lesions; (3) genetically autonomous EMZL characterized by accumulated lesions (e.g., t(11;18)(q21;q21)/API2–MALT1, *TNFAIP3*, *NOTCH1/2*, *MYD88*) and reduced reliance on the microenvironment; and (4) transformed diffuse large B‐cell lymphoma with high genomic complexity and loss of antigen dependence. Below the axis, major therapeutic strategies are positioned where they are most biologically appropriate: Pathogen‐directed antibiotics and involved‐site radiotherapy in the antigen‐dependent phase; anti‐CD20‐based chemoimmunotherapy (rituximab–chlorambucil, bendamustine–rituximab) in intermediate disease; and targeted agents (BTK inhibitors, lenalidomide–rituximab) and advanced immunotherapies (CAR‐T cells, bispecific antibodies) for genetically autonomous or transformed disease. Surrounding the continuum, red‐bordered boxes highlight key unmet needs: Robust predictive biomarkers to distinguish patients curable with conservative strategies from those requiring early systemic therapy, tools for early detection and prevention of histologic transformation, validated MRD/ctDNA assays for dynamic risk‐adapted management, and deeper microenvironmental profiling to inform future immunomodulatory approaches.

Figure 5 integrates these biological and clinical dimensions, conceptualizing EMZL as a dynamic spectrum ranging from immune‐driven, microenvironment‐dependent disease to genetically autonomous lymphoma, and mapping current therapeutic strategies and unmet needs across this continuum. Contemporary management, therefore, increasingly emphasizes precision, restraint, and biologically informed decision‐making to maximize durable disease control while minimizing treatment‐related morbidity.

## 7 Conclusion

EMZL represents a paradigmatic model of immune‐driven lymphomagenesis, in which chronic antigenic stimulation, a supportive microenvironment, and acquired genetic lesions cooperate to shape disease initiation, evolution, and therapeutic responsiveness. Its biology has translated into highly effective, low‐toxicity interventions that are curative, particularly, in early, antigen‐dependent disease.

At the same time, important challenges remain. The absence of validated predictive biomarkers, the clinical impact of histologic transformation, and the incomplete understanding of microenvironmental dynamics limit the full realization of precision medicine in this disease. Addressing these gaps will require integrated biological, molecular, and clinical approaches, coupled with prospective validation in well‐designed studies.

Ultimately, advancing care for patients with EMZL will depend on refining risk stratification, improving tools for dynamic disease monitoring, and developing strategies that anticipate progression or transformation, thereby enabling individualized treatment that balances long‐term disease control with preservation of quality of life.

## Author Contributions

**Santino Caserta**, **Mamdouh Skafi**, **Enrica Antonia Martino**, **Fortunato Morabito**, and **Massimo Gentile:** conceptualization. **Santino Caserta**, **Mamdouh Skafi**, **Enrica Antonia Martino**, **Francesco Mendicino**, **Maria Eugenia Alvaro**, **Ernesto Vigna**, **Antonella Bruzzese**, **Graziella D'Arrigo**, **Giovanni Tripepi**, and **Fortunato Morabito:** methodology. **Enrica Antonia Martino**, **Santino Caserta**, **Giovanni Tripepi**, **Fortunato Morabito**, **Nicola Amodio**, **Marco Fiorillo**, and **Massimo Gentile:** writing – original draft preparation. **Enrica Antonia Martino**, **Santino Caserta**, **Mamdouh Skafi**, **Eugenio Lucia**, **Virginia Olivito**, **Caterina Labanca**, **Francesco Mendicino**, **Maria Eugenia Alvaro**, **Fortunato Morabito**, and **Massimo Gentile:** writing, review, and editing. All authors have read and agreed to the published version of the manuscript.

## Funding

The authors have nothing to report.

## Ethics Statement

The authors have nothing to report.

## Conflicts of Interest

The authors declare no conflicts of interest.

## Data Availability

Data sharing not applicable to this article as no datasets were generated or analysed during the current study.
